# Renal metastasis from papillary thyroid carcinoma: A rare case report

**DOI:** 10.3389/fonc.2025.1597612

**Published:** 2025-09-19

**Authors:** Wen Huang, Zhenghui Hou, Yuxi Lin, Qianrong Bai, Menghui Yuan, Peng Yuan

**Affiliations:** ^1^ Department of Nuclear Medicine, Tangdu Hospital, Fourth Military Medical University, Xi’an, China; ^2^ Medical School of Yan'an University, Yan’an, China

**Keywords:** thyroid cancer, renal metastasis, lung metastasis, bone metastasis, radioactive iodine-125 seed

## Abstract

This article presents a rare case of renal metastasis from papillary thyroid carcinoma (PTC) with concurrent lung and bone metastases. Through a retrospective analysis of the patient’s diagnostic and treatment process, we aim to contribute to the development of precise treatment strategies for such rare cases, thereby improving patient prognosis.

## Introduction

Thyroid cancer is one of the most prevalent malignancies in the endocrine system and represents a significant public health challenge. Its incidence has shown a rapid upward trend over the past few decades. Thyroid cancer is classified into four main pathological types: Papillary Thyroid Carcinoma (PTC), Follicular Thyroid Carcinoma (FTC), Anaplastic Thyroid Carcinoma (ATC), and Medullary Thyroid Carcinoma (MTC) (1). Among these, PTC is the most common, accounting for 90% of thyroid tumors, while FTC constitutes 5% to 10% of thyroid tumors. Although many PTC patients have a favorable prognosis with a 5-year survival rate of 95%, 10%-20% of well-differentiated thyroid cancers are associated with distant metastases. Follicular thyroid cancer most commonly metastasizes to the lungs and bones, whereas papillary thyroid cancer is less frequently associated with bone metastasis. For patients with radioactive iodine-refractory locally advanced or metastatic differentiated thyroid cancer, surgical resection or stereotactic body radiotherapy is superior to systemic therapy (2). Two oral multi-target tyrosine kinase inhibitors, sorafenib tosylate and lenvatinib (3), are available; however, survival rates remain unfortunately low, necessitating new research approaches utilizing novel technologies (4).

**Figure 1:**
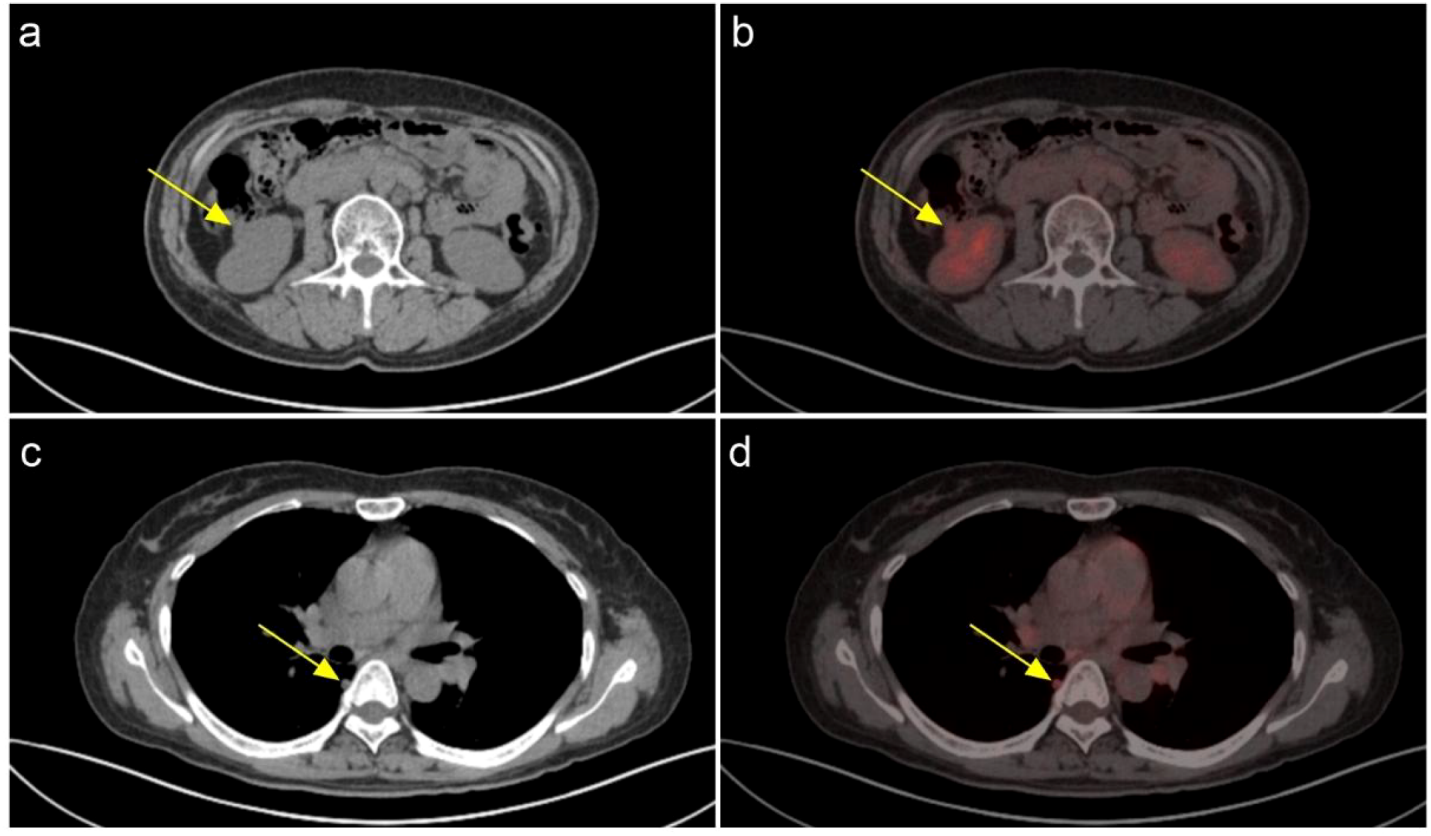
A soft tissue nodule is present in the lower pole cortex of the right kidney, with increased glucose metabolism. A neoplastic lesion cannot be ruled out. **(A)** CT **(B)** PET-CT; Multiple solid minute nodules are present in both lungs, with the largest one located paraspinally in the dorsal segment of the lower lobe of the right lung. **(C)** CT **(D)** PET-CT.

**Figure 2:**
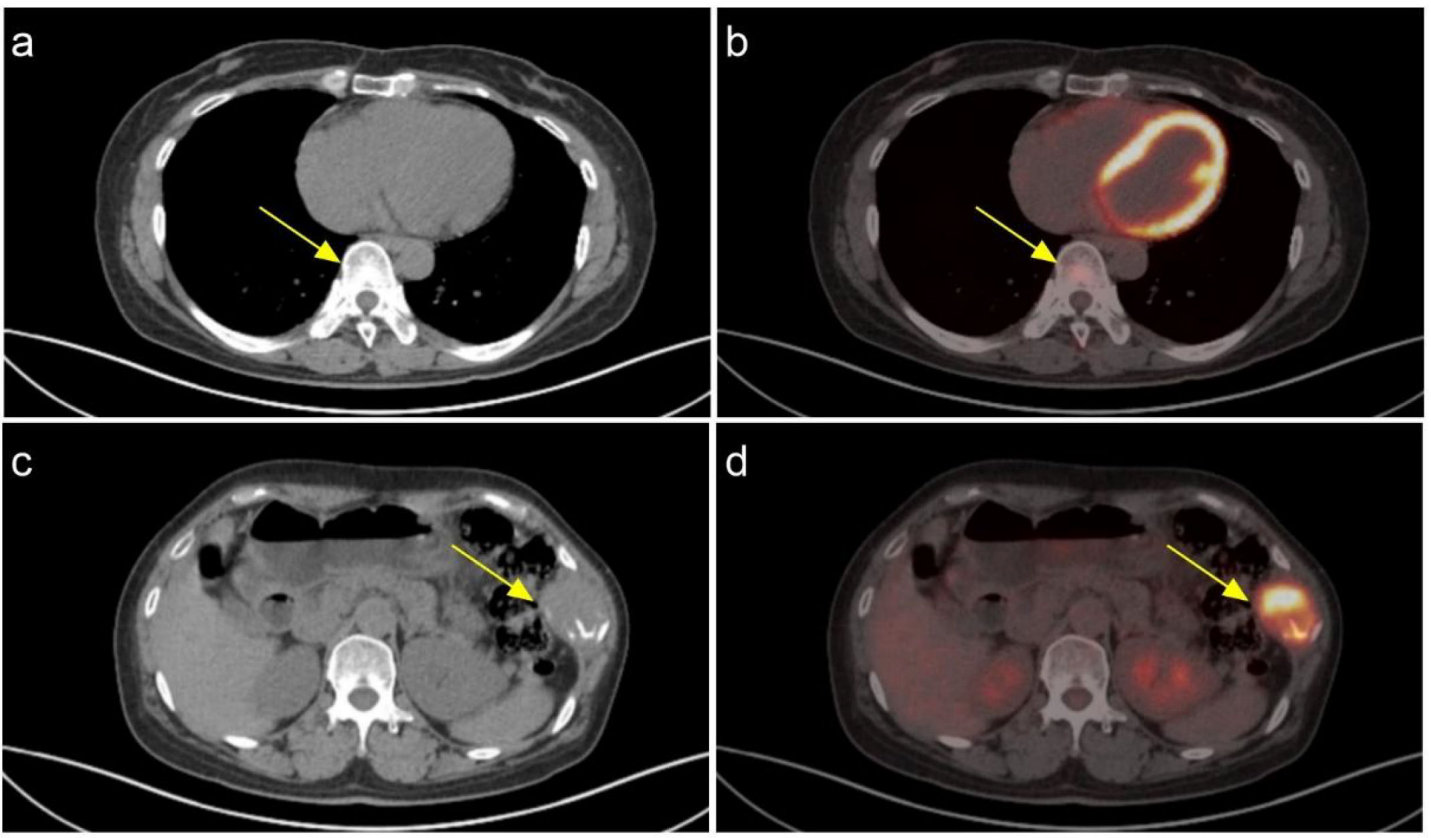
The increased glucose metabolism in the eighth thoracic vertebra is consistent with the imaging manifestations of a metastatic tumor. **(A)** CT **(B)** PET-CT; The increased glucose metabolism in the 9th rib on the left side is consistent with the radiological manifestations of a metastatic tumor. **(C)** CT **(D)** PET-CT.

**Figure 3:**
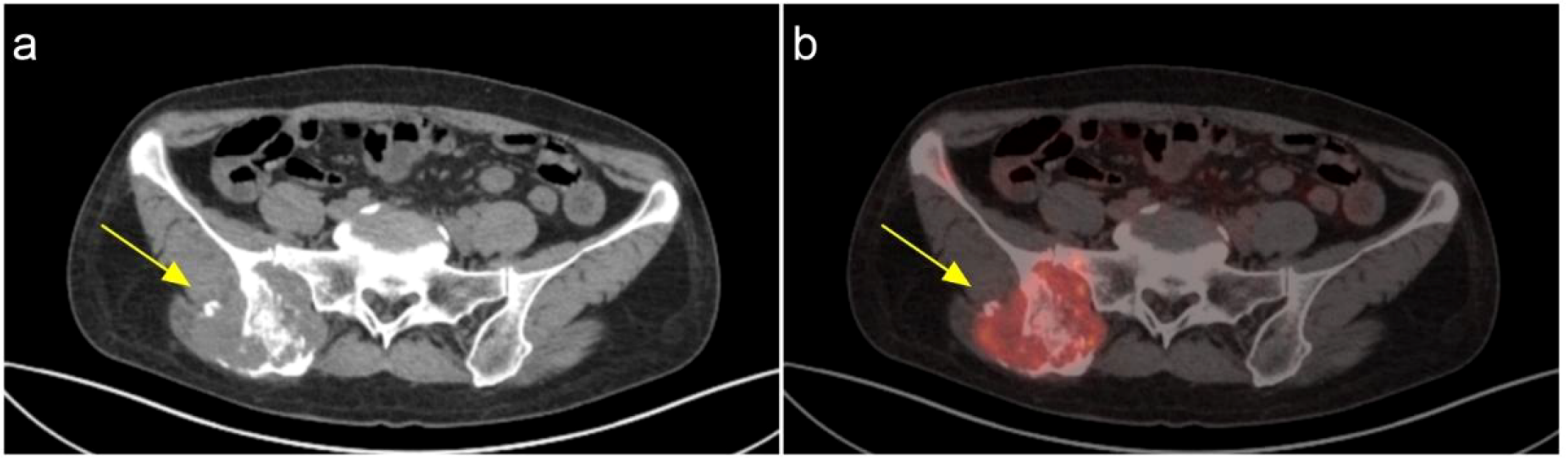
The iliac bone on the right side demonstrates bone destruction accompanied by the formation of a surrounding soft tissue mass, with involvement of the adjacent sacroiliac joint and iliac wing of the sacrum. Increased glucose metabolism is observed, which is consistent with the radiological manifestations of metastatic tumors. **(A)** CT **(B)** PET-CT.

A 48-year-old female patient presents with a chronic oncological condition. She underwent a partial nephrectomy of the right renal space-occupying lesion in December 2024 after a tumor in the right kidney (**Figures 1A, B**) was detected during a routine physical examination. In her past medical history, the patient underwent a subtotal thyroidectomy in 2012 due to papillary thyroid carcinoma on the left side, with postoperative pathology confirming the diagnosis. Additionally, the patient exhibits multiple bony metastases (**Figures 2**, **3**), and pulmonary metastases (**Figures 1C, D**) throughout her body. She has no history of other diseases, no family history of cancer, and no abnormal psychosocial history.

**Figure 4:**
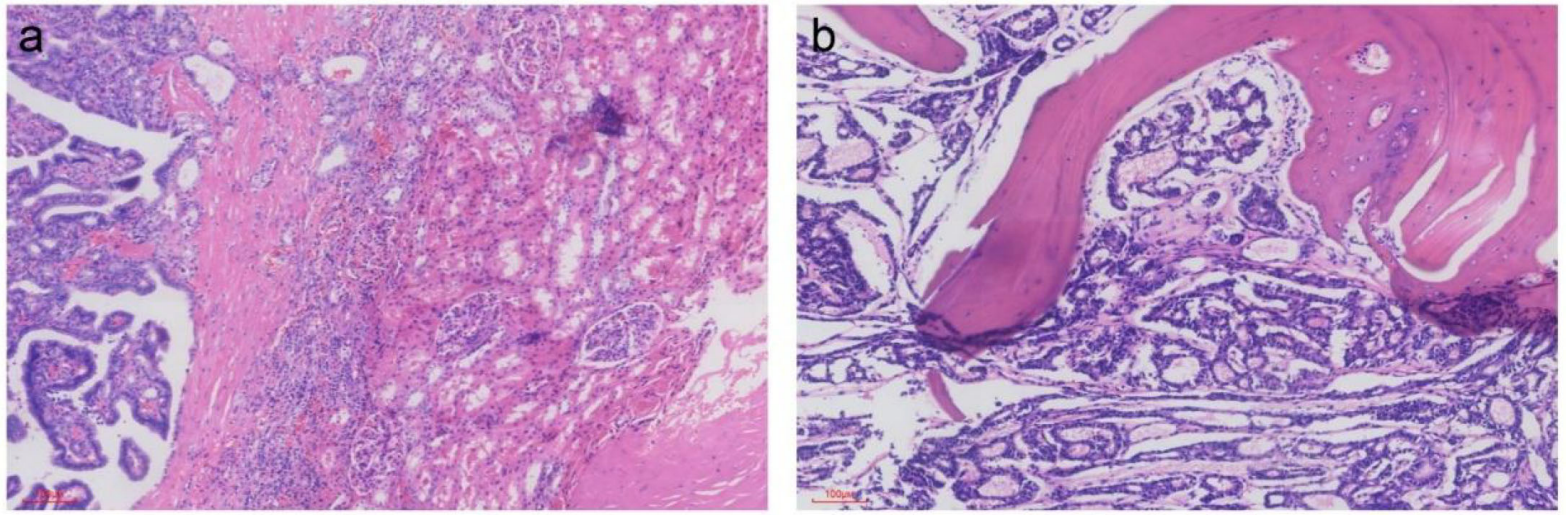
Pathology: (Right kidney tumor): Metastatic thyroid papillary carcinoma was found within the renal tissue. **(A)** (Right iliac bone lesion): Glandular tubular structures were identified in the submitted coagulated tissue. Based on histological, immunohistochemical, and clinical findings, the findings suggest metastatic thyroid tumor. **(B)** ([HE]; magnification, ×100).

**Figure 5:**
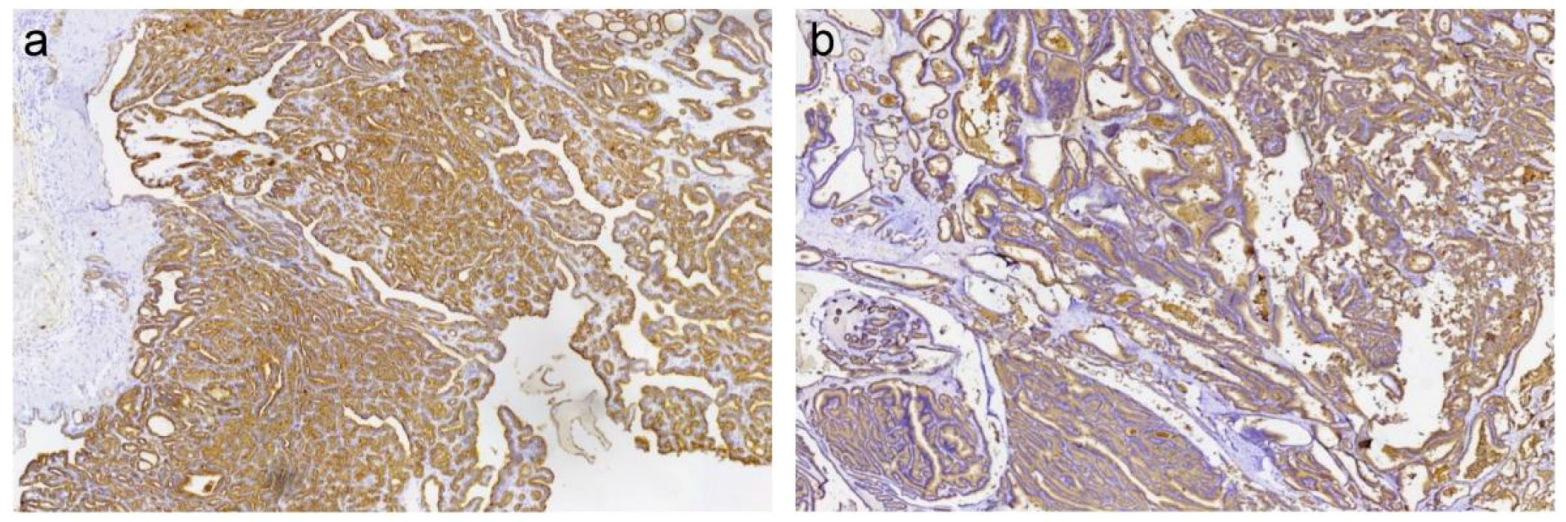
Immunohistochemical staining shows positive expression of Thyroglobulin (Tg) in the tumor cells (brown). Positive signals are localized in the cytoplasm. (Right kidney tumor) **(A)**; (Right iliac bone lesion) **(B)**;.

In October 2024, the patient presented to the orthopedic department with intermittent low back pain and was diagnosed with suspected secondary malignant tumors in multiple bones upon examination. In October 2024, the patient underwent a biopsy of bone destruction in the right pelvis, and the postoperative pathology indicated bone metastasis from thyroid cancer (**Figures 4B**, **5B**). Subsequently, the patient received an exploratory curettage of the right pelvic tumor, microwave ablation, and bone cement filling. In November 2024, the patient underwent computed tomography (CT) - guided implantation of radioactive ¹²^5^I seeds into the soft tissue of the left chest wall and thoracic vertebrae. Shortly thereafter, the patient also underwent laparoscopic partial nephrectomy of the right kidney, with postoperative pathology revealing metastatic foci of papillary thyroid carcinoma within the right renal tissue (**Figures 4A**, **5A**).

Physical Examination: No bulging was observed in the bilateral renal regions, and no tenderness or percussion pain was noted. There was no tenderness along the course of the bilateral ureters, and no tenderness was detected in the bladder area.

## Discussion

Papillary thyroid carcinoma primarily metastasizes through the lymphatic system. Tumor cells can migrate via lymphatic vessels to cervical lymph nodes, including those in the central and lateral neck regions. Hematogenous spread to distant organs is less common, and it is even rarer for distant metastases to be the initial presenting symptom. Among the less common distant metastatic sites, the lungs are included. The cervical lymph nodes are the most frequent sites of metastasis (3). In contrast, follicular thyroid carcinoma mainly spreads through the bloodstream. Once cancer cells enter the circulatory system, they can metastasize to distant organs. The most common distant metastatic sites are the lungs (5) and bones (6), with other possible sites including the liver (7) and adrenal glands (8). Medullary thyroid carcinoma also primarily metastasizes through the lymphatic system, particularly to the central and lateral cervical lymph nodes, but can also spread hematogenously. Distant metastases may involve organs such as the lungs (9) and liver (10). Common primary tumors that metastasize to the kidneys include lung cancer (11), breast cancer (12), liver cancer (13) colorectal cancer (14), and malignant melanoma (15).

Here, we present a rare case of renal metastasis from papillary thyroid carcinoma. The patient presented with concurrent metastases involving the pulmonary parenchyma, skeletal system, and kidneys. Given the patient’s condition, we prioritized symptomatic treatment. The patient experienced a right gluteal mass with worsening pain, accompanied by right iliac bone destruction and a surrounding soft tissue mass. Multi-Disciplinary Treatment discussion clinical manifestations, physical examination, and imaging findings, surgical intervention was deemed appropriate. The patient subsequently underwent exploratory surgery with curettage, microwave ablation, and bone cement augmentation for the right pelvic lesion. Concurrently, due to identified destructive changes in the left 9th rib with associated soft tissue mass and radiographic evidence of metastatic involvement in the T8 vertebral body, CT-guided ^125^I seed implantation was performed targeting the thoracic wall soft tissue and spinal lesions. Subsequently, a laparoscopic partial nephrectomy was performed for a right renal mass to remove the kidney lesion and inhibit disease progression. Notably, as the patient had not undergone previous total thyroidectomy, Multi-Disciplinary Treatment discussion contraindicated completion thyroidectomy, thereby precluding subsequent radioactive iodine therapy. Postoperative management includes continued TSH-suppressive therapy and molecular targeted therapy with lenvatinib. During the 9-month postoperative follow-up, the patient remained clinically stable. With TgAb being negative, the serum non-stimulated thyroglobulin level decreased from 45.4 ng/mL to 4.75 ng/mL. The patient exhibited stable vital signs and maintained good neurological function.

## Data Availability

The original contributions presented in the study are included in the article/supplementary material. Further inquiries can be directed to the corresponding author.
